# Relationship between human leukocyte antigen (HLA)-DQA1*0102/HLA-DQB1*0602 polymorphism and preeclampsia

**Published:** 2017-09

**Authors:** Mahmoud Mohammadi, Touraj Farazmandfar, Majid Shahbazi

**Affiliations:** *Medical Cellular and Molecular Research Center, Golestan University of Medical Sciences, Gorgan, Iran.*

**Keywords:** Preeclampsia, HLA-DQ, Genotyping

## Abstract

**Background:**

Preeclampsia is a condition associated with systemic disorders in the mother and the fetus. However, the exact causes of preeclampsia are unknown, but several genetics and environmental factors play role in development of this disease. Major histocompatibility complex role is very important during pregnancy through which the fetus is not rejected by mother’s immune system.

**Objective:**

In this study, we investigated the relationship of the human leukocyte antigen (HLA)-DQA1*0102/HLA-DQB1*0602 polymorphism with preeclampsia.

**Materials and Methods:**

Genomic DNA of 181 pregnant women with a history of preeclampsia as the case group and 228 pregnant women with no history of preeclampsia as the controls were extracted. The HLA-DQA1*0102/HLA-DQB1*0602 polymorphisms of all DNA samples were identified by the SSP-PCR method. Frequencies difference of variables between case and control groups were calculated by Chi-square test. The ethnic origin of the participants in this study was extracted from their medical records.

**Results:**

There was a significant association between preeclampsia and Sistani ethnic group (p=0.031). Moreover, there was a significant association between preeclampsia and frequencies of allele HLA-DQB1*0602 (p<0.001), and genotypes of heterozygote (+0102/-0602) (p<0.001) and negative homozygote (-0102/-0602) (p=0.005). There also was an association between allele HLA-DQB1*0602 and preeclampsia in Fars ethnic group (p=0.028).

**Conclusion:**

It seems that immune incompatibility may have an important role in preeclampsia predisposition. According to our results, the lack of locus HLA-DQB1*0602 may be a risk factor for preeclampsia.

## Introduction

Preeclampsia is one of the most common complications during pregnancy which is considered as one of the main three causes of death among pregnant women throughout the world. Despite many studies, etiology of this disease is not fully clear (1-3). However, there is no reliable criterion for early detection of preeclampsia yet; But there are numerous clinical, biophysical, and biochemical tests to identify women at risk for preeclampsia which their results demonstrate the low predictive value in early diagnosis of preeclampsia. Various factors such as oxidative stress, endoplasmic reticulum stress, immunological factors, and vascular endothelial activation are involved in the pathogenesis of preeclampsia that for each of which corresponding genetics factors can be found (4-7). Therefore, many genetic studies have been conducted in this area. Additionally, many studies have been performed on the impact of environmental and epistasis factors in preeclampsia (6, 7).

Hence, we can say that preeclampsia is a multifactorial disease and no individual gene can be introduced as the key factor. So far, many candidate genes associated with preeclampsia have been studied. These genes act in different specified pathophysiological ways of the disease (7, 8). Like other genetically complex diseases, the results of studies on considered genes have been inconsistent, so that no global susceptibility gene has been set for this disease yet. Some differences are because of the diversity in the populations but much of it is likely due to the small population of most candidate genes studies. This led to the identification of genes with small effect. Major histocompatibility complex (MHC) play a very important role during pregnancy through which the fetus is not rejected by mother’s immune system (9). 

Human leukocyte antigens (HLA) play a major role in transplantation and pregnancy from the formation of gamete to the completion of the development. The alloimmune HLA-DQ sharing between the embryo recipient (female partner) and the sperm provider (male partner) leads to interfering with implantation and loss of fertility. This sharing generally requires continuous exposure of the fetus to the host's uterine natural killer cells that are active enough and may lead to damage to trophoblast (9-12). Therefore, MHC complex plays a very important role during pregnancy through which the fetus is not rejected by mother’s immune system (9). 

HLA-DQ is a cell surface receptor protein found on the surface of antigen-presenting cells. HLA-DQ is a MHC class II αβ heterodimer. Alpha and beta chains are coded by HLA-DQA1 and HLA-DQB1 respectively and these two loci are in close proximity to each other on chromosome 6p21.3. These loci have twenty-five alleles and vary in different populations. The beta subunit is more variable than α subunit. The variants coded by HLA-DQ genes are created as a result of removing a part of the third intron of the HLA-DQ gene sequence and also single nucleotide polymorphisms. HLA-DQ is one of the several antigens involved in the rejection of organ transplants (12). The defect in cellular and humoral immune system increases the cytotrophoblast invasion. This defect in HLA can play a role in mother's immune system which its interference with fetal development, and eventually leads to immune incompatibility. Therefore, studying MHC in preeclampsia seems to be necessary. 

According to these facts, we aimed to investigate the association of the two most common alleles of the locus HLA-DQ with preeclampsia.

## Materials and methods

**Subjects**

In this case-control study, alleles of HLA-DQA1*0102 and HLA-DQB1*0602 were genotyped in 181 pregnant women with preeclampsia history as the case group and 228 healthy pregnant women with no history of preeclampsia as the control group. Our participants were selected from pregnant women referred to Shahid Sayad Shirazi Hospital, Golestan University of Medical Sciences, Gorgan, Iran, from March 2010 to May 2013.

Inclusion criteria for subjects with preeclampsia was included high blood pressure (systolic and diastolic blood pressure greater than or equal to 140 and 90 mmHg respectively) and 24 hr proteinuria >300 mg and/or +1 without kidney diseases and infection. Inclusion criteria for control group was included a healthy pregnancy with no history of preeclampsia. Exclusion criteria for both groups were included inflammatory, autoimmune and chronic or infectious diseases such as Hepatitis B. The ethnic origin of the participants in the study was extracted from their medical records.

**Genotyping**

DNA was extracted from 5 ml whole blood samples by a standard method as described previously (13). Polymorphisms of HLA DQA1*0102 and HLA DQB1*0602 were genotyped by Sequence Specific Primer-Polymerase Chain Reaction (SSP-PCR) method using specific primers (Table I). PCR reactions were performed using PCR master mix (Ampliqon, Copenhagen, Denmark) with 100 nanograms of DNA and 5 picomol of each primer in a thermal cycler (Bio-Rad, Munich, Germany). The Glyceraldehyde 3-phosphate dehydrogenase (GAPDH) cDNA was also amplified as a PCR internal control. 

**Figure 1:**
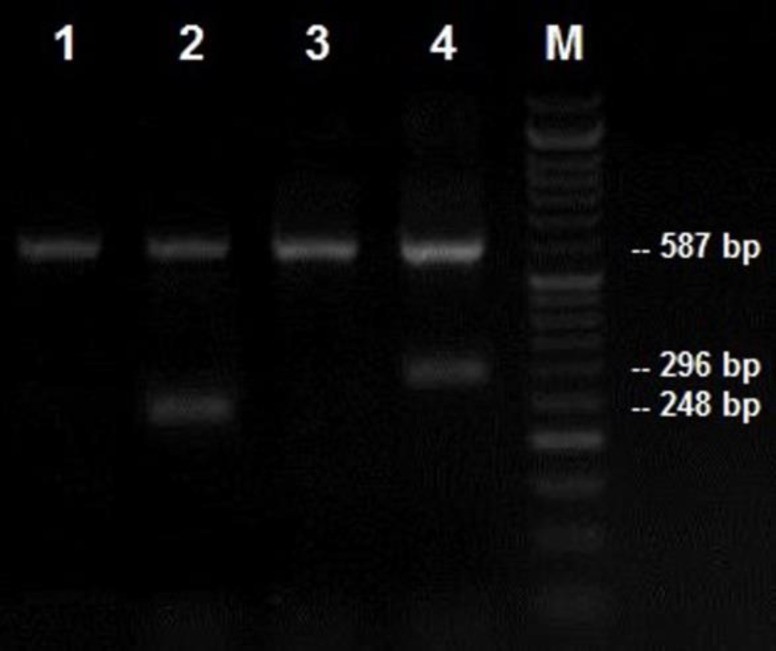
Electrophoresis pattern of PCR products for detection of HLA-DQA1*0102/HLA-DQB1*0602 polymorphism. Band 248 bp presents allele HLA-DQA1*0102. Therefore, column 1 is a negative sample, and column 2 is a positive sample. Band 296 bp presents allele HLA-DQA1*0602. Therefore, column 3 is a negative sample, and column 4 is a positive sample. M, DNA marker; Band 248 bp presents the amplified GAPDH, as control bond

The PCR protocol included one initial denaturation at 95^o^C for 4 min, 10 cycles (15 sec at 95^o^C, 50 sec at 65^o^C and 40 sec at 72^o^C) followed by 25 cycles (20 sec at 95^o^C, 50 sec at 58^o^C and 50 sec at 72^o^C), with a final extension at 72^o^C for 5 min. The PCR products, after staining with safe stain, were subjected to electrophoresis and photographed under a UV transilluminator (UVITEC, Cambridge, UK) as described previously (14). The two alleles of HLA-DQA1*0102 and HLA-DQB1*0602 are identified by lack of a portion of intron 3. Therefore, HLA-DQ alleles could be detected by product size band; 248 base paired (bp) for the allele HLA-DQA1*0102, and 296 bp for allele HLA-DQB1*0602 (Figure 1).

**Ethical consideration**

All participants in this study were aware of the study details, and the relevant written informed consent was signed by all individuals. The Ethics Committee of Golestan University of Medical Sciences approved the study for collecting data and performing the project (IR.GOUMS.REC.1395.14).

**Statistical analysis**

Frequencies difference between case and control groups was calculated by Chi-square test. Data was analyzed by SPSS (Statistical Package for the Social Sciences, version 16, IBM, Armonk, NY, USA). The level of statistical significance was defined as p<0.05.

## Results

In this study, the control and case groups were divided into three age category: 16-25, 26-35, and 36-45 yr old, and no significant association was observed between these age groups and preeclampsia (Table II). Moreover, the present study is composed of four groups of Fars, Turkmen, Sistani and other ethnic groups, and there was a significant association between Sistani folk and preeclampsia among ethnic groups. (p=0.031) (Table II). The genotypic and allelic distributions in the case and control groups are presented in table III. 

The allelic frequencies of HLA-DQA1*0102 and HLA-DQB1*0602 in case group were 61.9% and 75.7% respectively, and in control group were 59.6% and 92.1% respectively. The statistical analysis showed that there was no significant difference between the control group and the patient group in allelic distribution of HLA-DQA1*0102. However, a significant association was observed between low frequency of allele HLA-DQB1*0602 and preeclampsia (p<0.001). In addition, according to table III, the higher frequency of two genotypes: heterozygote (+0102/-0602) (p<0.001) and negative homozygote (-0102/-0602) (p=0.005), were significantly correlated with preeclampsia. More analysis was performed for each locus in terms of ethnicity, and there was no significant association between locus HLA-DQA1*0102 and preeclampsia (Table IV). But in locus HLA-DQB1*0602, there was an association between locus and preeclampsia in Fars ethnic group (p=0.028) (Table V).

**Table I:** The used primers in this study

**Gene**	**Primers 5ʹ - 3ʹ**		**Band size (bp)**	**Annealing temperature (** ^o^ **C)**	**Genbank accession** **number**
HLA-DQA1*0102	Forward	CTGACCACGTTGCCTCTTGT	248	58	NG_032876.1
	Reverse	ATTGGTAGCAGCGGTAGAGTT			
HLA-DQB1*0602	Forward	TCCCCGCAGAGGATTTCGTGT	294	57	NG_029922.1
	Reverse	TGCAGGGCGACGACGCTCACCTCTCC			
GAPDH	Forward	GATGCTGGCGCTGAGTACGTCG	587	65	NG_007073.2
	Reverse	GAGGAGACCACCTGGTGCTCAG			

**Table II:** Comparison of the frequency of preeclampsia cases and healthy controls based on age and ethnicity

**Variables**			**Case group**	**Control group**	**OR (CI 95%)**	**p-value**
Maternal age (yr)						
		16-25	38 (3.3)	42 (3.9)	1.39 (0.83-2.30)	0.241
		26-35	101 (94.5)	155 (95.6)	Ref	
		36-45	26 (2.2)	31 (0.5)	1.29 (0.72–2.31)	0.453
Ethnic groups						
	Fars		94 (51.9)	105 (46.1)	Ref	
	Turkman		31 (17.1)	26 (11.4)	1.33 (0.73-2.41)	0.346
	Sistani		46 (25.5)	85 (37.3)	0.60 (0.38-0.95)	0.031
	Others		10 (5.5)	12 (5.2)	0.93 (0.38-2.25)	1.000

Comparison analysis was done by Chi-square test.

Data presented as n (%)

N: number

OR: odds ratio

CI: confidence interval

Ref: reference.

**Table III:** The allelic and genotypic distribution of HLA-DQA1*0102/HLA-DQB1*0602 polymorphism in case group and control group

**Alleles**			**Case group**	**Control group**	**OR (CI 95%)**	**p-value**
HLA-DQA1*0102						
		Positive	112 (61.9)	136 (59.6)	Ref	
		Negative	69 (38.1)	92 (40.4)	0.91 (0.61-1.35)	0.684
HLA-DQB1*0602						
	Positive		137 (75.7)	210 (92.1)	Ref	
	Negative		44 (24.3)	18 (7.9)	3.74 (2.07-6.75)	< 0.001
Genotypes (0102/0602)						
		Positive/Positive	91 (50.3)	129 (56.6)	Ref	
		Positive/Negative	21 (11.6)	7 (3.1)	4.25 (1.73–10.43)	< 0.001
		Negative/Positive	46 (25.4)	81 (35.5)	0.80 (0.51-1.26)	0.364
		Negative/Negative	23 (12.7)	11 (4.8)	2.96 (1.38-6.38)	0.005

Comparison analysis was done by Chi-square test.

Data presented as n (%)

N: number

OR: odds ratio

CI: confidence interval

Ref: reference.

**Table IV:** The association analysis of allele HLA-DQA1*0102 with preeclampsia in ethnic groups of Golestan province in northern Iran

**Ethnic group**	HLA-DQA1*0102		**OR (CI 95%)**	**p-value**
	**Negative**	**Positive**		
Persian				
Control	35 (33.3)	70 (66.7)	Ref	0.146
Case	41 (43.6)	53 (56.4)	1.55 (0.87-2.75)	
Torkman				
Control	14 (53.9)	12 (46.1)	Ref	0.792
Case	15 (48.4)	16 (51.6)	0.80 (0.28-2.28)	
Sistani				
Control	31 (36.5)	54 (63.5)	Ref	0.850
Case	18 (39.1)	28 (60.9)	1.12 (0.53-2.34)	
Other groups				
Control	3 (25.0)	9 (75.0)	Ref	0.651
Case	4 (40.0)	6 (60.0)	2.00 (0.32-12.30)	

Comparison analysis was done by Chi-square test.

Data presented as n (%)

N: number

OR: odds ratio

CI: confidence interval

Ref: reference.

**Table V:** The association analysis of allele HLA-DQB1*0602 with preeclampsia in ethnic groups of Golestan province in northern Iran

**Ethnic group**	HLA-DQB1*0602		**OR (CI 95%)**	**p-value**
	**Negative**	**Positive**		
Fars				
Control	10 (9.5)	95 (90.5)	Ref	0.028
Case	20 (21.3)	74 (78.7)	2.57 (1.13-5.82)	
Torkman				
Control	4 (15.4)	22 (84.6)	Ref	0.741
Case	6 (19.4)	25 (80.6)	1.32 (0.33-5.30)	
Sistani				
Control	7 (8.2)	78 (91.8)	Ref	0.151
Case	8 (17.4)	38 (82.6)	2.35 (0.79-6.95)	
Others				
Control	4 (33.3)	8 (66.7)	Ref	1.000
Case	3 (30.0)	7 (70.0)	0.86 (0.14-5.23)	

Comparison analysis was done by Chi-square test.

Data presented as n (%)

N: number

OR: odds ratio

CI: confidence interval

Ref: reference.

## Discussion

The preeclampsia is one of the most common pregnancy complications and the leading cause of death in mother and fetus. Abortive attempts have been done to identify the etiology of this disease but it is not fully clear yet. Preeclampsia is one of the main three causes of death among pregnant women around the world and is raised as a global problem of women's health by the World Health Organization (1, 2). Despite many hypotheses for preeclampsia, it is thought that this disease is caused by defects in vascular endothelial cell function and reduced blood supply to the placenta (3, 4). Many hypotheses have been proposed about the factors increasing the susceptibility to preeclampsia including the impact of immunological factors, coagulation disorders, nutritional factors, and increased production of reactive oxygen spices (5, 6, 15, 16). Nowadays, it is entirely clear that preeclampsia is a genetically complex disorder (7). 

In fact, preeclampsia follows Mendelian inheritance pattern only in a small number of families including the rare single-gene deletions and the influential mutation. But for the majority of the population, preeclampsia is a complex genetic disorder. This means that a large number of gene variants are involved in preeclampsia so that each of these genes may have a small effect alone, but their cumulative effect would be devastating. Different subsets of this disease to be likely associated with different gene variants along with intrauterine growth restriction (7, 8). 

Therefore, preeclampsia may be a multifactorial disease in which the interaction among different genes or the interaction between these genes with environmental factors will determine the susceptibility to it. As our results in table II showed, there is a significant association between preeclampsia and some of the ethnic groups such as Sistani resident in northern Iran. However, this association may be due to a high pregnancy rate among these ethnic groups. In this study, the frequencies of *HLA* gene polymorphisms, HLA-DQA1*0102, and HLA-DQB1*0602 were investigated among healthy subjects and patients with preeclampsia. The results of this analysis showed that there was a significant difference in the frequency of only locus HLA-DQB1*0602 in preeclampsia group compared to control subjects (Table III). 

Therefore, it is clear that the lack of allele HLA-DQB1*0602 may be an effective factor in preeclampsia. Moreover, this allele has a significant high frequency in Fars ethnic group. Therefore, it can be considered as a predisposing allele associated with preeclampsia. The results of Humphrey *et al* study on deletion polymorphism of *HLA-G* gene in women with preeclampsia showed that there was no significant correlation between the risk of preeclampsia in pregnancy and HLA-G genotypes (17). While Hunt *et al* carried out study on HLA-G expression in fetal cytotrophoblast cells and introduced *HLA* genes as the main genetic factor in immune responses that play an important role in reproduction (18). Furthermore, Pfeiffer *et al* study showed an increase in the frequency of HLA-G*01013 and HLA-G*0105N carriers in Recurrent Spontaneous Abortion (RSA) group compared to the control group, while Aldrich *et al* study showed an increase in HLA-G*0104 or 0105 in RSA group (19, 20). 

The Mao *et al* study evaluated the relationship between *HLA* with high blood pressures occurring during pregnancy but no association was found in frequencies of HLA-DQB1 and HLA-DQA1 alleles between two groups. But they concluded that HLA-DQB1*0503 may be a marker in high blood pressures in pregnancy (21). In another study, Honda *et al* investigated the relationship of HLA-DQB1 and HLA-DPB1 to severe pregnancies and their data suggested that women who have the allele HLA-DQB1*04 might be susceptible to preeclampsia (22). Moreover, Ooki *et al* carried out an experiment in order to probe HLA class II alleles among couples with severe preeclampsia. Their findings indicated a significant relationship between HLA class II types and high blood pressure (23). 

In this study, one of the major points may be multi-ethnic participants. However, different ethnic groups in Golestan province influence the final result. Therefore, in order to better evaluate the effect of the two alleles on this disease, it is suggested to increase the population to be able to judge confidently in this regard. Considering the few studies have been performed on association assessment of HLA-DQ with preeclampsia, thus results of this study can be informative to analysis effect of MHC on pregnancy.

## Conclusion

It seems that the lack of allele HLA-DQB1*0602 is a significant factor for preeclampsia. Therefore, deletion variant of this polymorphism may be a risk factor for preeclampsia and can be further considered.
